# Supplementary material for: Evolutionary dynamics of transposable elements in bdelloid rotifers
Source: eLife. 2021 Feb 5;10:e63194. doi: 10.7554/eLife.63194 (PMC7943196; doi:10.7554/eLife.63194)
Supplement: Figure 2—source data 4. [file elife-63194-fig2-data4.docx]

**Figure 2—source data 5.** Model output for phylogenetic models testing for significant shift in TE frequency on bdelloid stem branch compared to background (**Table 1**), and MEDUSA-like test for significant shifts in rate of TE evolution on phylogenetic tree of bdelloids, monogononts and outgroups (**Table 2**).

**Table 1.** No evidence for a significant decrease in TE frequency on bdelloid stem branch.

|  | Null model | | Shift model | | |  |  |
| --- | --- | --- | --- | --- | --- | --- | --- |
|  | Lambda^1^ | Log L | Lambda |  | Log L | Log L ratio test | |
|  |  |  | Background | Bdelloid stem |  | Chi-square^2^ | *P* |
| DNA transposons | 0.0117 | -72.3 | 0.0118 | 0 | -72.0 | 0.73 | 0.39 |
| Rolling circles | 0.0017 | -15.1 | 0.0017 | 0.0017 | -15.1 | 0.00 | 0.96 |
| Penelope | 0.0034 | -36.1 | 0.0034 | 0.0034 | -36.1 | 0.00 | 1.00 |
| LTRs | 0.0145 | -78.6 | 0.0146 | 0.0023 | -78.5 | 0.20 | 0.65 |
| LINEs | 0.0434 | -111.0 | 0.0408 | 0.3001 | -110.0 | 2.02 | 0.16 |
| SINEs | 0.0012 | -4.8 | 0.0012 | 0.0012 | -4.8 | 0.00 | 0.96 |
| class I | 0.106 | -137.4 | 0.1011 | 0.5895 | -136.7 | 1.40 | 0.24 |
| class II | 0.0175 | -84.3 | 0.0176 | 0 | -83.9 | 0.77 | 0.38 |
| All known | 0.1248 | -142.2 | 0.1193 | 0.6796 | -141.5 | 1.34 | 0.25 |

^1^ Rate of Brownian evolution

^2^ Twice the difference in Log L (1 degree of freedom)

**Table 2.** Evidence for a lower rate of TE evolution within bdelloid clade.

|  |  | Log L | N params | AICc^2^ | Lambda 1 | Lambda 2 | Lambda 3 |
| --- | --- | --- | --- | --- | --- | --- | --- |
| Class I | no-shift model | -137.37 | 2 | 278.96 | 0.11 |  |  |
|  | shift 1 (bdelloids)^1^ | -102.67 | 3 | 211.77 | 0.11 | 0.02 |  |
|  | shift 2 (*P. laevis*) | -94.91 | 5 | 200.95 | 0.11 | 0.05 | 28.89 |
| Class II | no-shift model | -84.31 | 2 | 172.83 | 0.02 |  |  |
|  | shift 1 (within *A. steineri*) | -75.11 | 3 | 156.66 | 0.02 | 1.00E-08 |  |
|  | shift 2 (*C. gigas*) | -69.32 | 5 | 149.78 | 0.02 | 1.00E-08 | 20.67 |

^1^ Clade shifted model fitted: lower lambda within bdelloids. N.B. The method distinguishes between clade shifts and branch shifts: a clade shift fits a different lambda for the entire clade, meaning a lower rate of evolution within the whole clade (less variability in the trait); a branch shift fits a separate lambda for a single branch, for example as expected with a shift to higher or lower mean trait value for the entire clade.

^2^ Corrected Akaike Information Criterion
