# Supplementary material for: Evolutionary dynamics of transposable elements in bdelloid rotifers
Source: eLife. 2021 Feb 5;10:e63194. doi: 10.7554/eLife.63194 (PMC7943196; doi:10.7554/eLife.63194)
Supplement: Figure 2—source data 5. [file elife-63194-fig2-data5.docx]

**Figure 2—source data 6.** Posterior mean and 95% credible intervals (CI) for the effect of desiccation ability (model levels: nondesiccating = 0, desiccating = 1) on (log) TE load (defined as the proportion [%] of genome accounted for by each TE classification) from a MCMCglmm Gaussian model.

**Table 1.** No evidence for an effect of desiccation on TE load within bdelloids.

| Test | Fixed effects | | | | Random effects | |
| --- | --- | --- | --- | --- | --- | --- |
|  | Intercept (95% CI) | pMCMC | ‘is.desiccating’ (95% CI) | pMCMC (FDR adjusted) | ID (phylogeny) (95% CI) | Residual (95% CI) |
| DNA transposons | 0.59 (-0.22, 1.51) | 0.15 | 0.27 (-0.27, 0.83) | 0.15 (0.49) | 0.33 (0.12, 0.63) | 0.00 (0.00, 0.01) |
| Rolling circles | -1.56 (-2.13, -1.02) | <5e-04 | 0.31 (-0.05, 0.75) | 0.10 (0.49) | 0.10 (0.00, 0.33) | 0.08 (0.04, 0.12) |
| PLEs | -0.71 (-1.78, 0.20) | 0.15 | 0.25 (-0.37, 0.89) | 0.40 (0.60) | 0.42 (0.11, 0.86) | 0.01 (0.00, 0.01) |
| LTRs | -0.11 (-2.40, 2.07) | 0.92 | -0.25 (-0.86, 0.49) | 0.43 (0.60) | 1.44 (0.01, 3.21) | 0.04 (0.00, 0.09) |
| LINEs | 0.23 (-0.90, 1.61) | 0.69 | -0.21 (-1.04, 0.57) | 0.58 (0.63) | 0.69 (0.25, 1.21) | 0.00 (0.00, 0.01) |
| Satellite | -3.56 (-5.14, -1.79) | <0.01 | -0.53 (-1.75, 0.71) | 0.34 (0.60) | 0.97 (0.00, 2.97) | 0.69 (0.33, 1.12) |
| Simple | -0.28 (-1.23, 0.62) | 0.53 | 0.41 (-0.19, 1.00) | 0.15 (0.49) | 0.37 (0.10, 0.79) | 0.01 (0.00, 0.02) |
| Low complexity | -1.84 (-2.50, -1.15) | <5e-04 | 0.46 (-0.00, 0.89) | 0.05 (0.49) | 0.21 (0.08, 0.39) | 0.00 (0.00, 0.01) |
| Unclassified | 2.94 (1.83, 4.10) | <5e-04 | 0.25 (-0.50, 0.96) | 0.46 (0.60) | 0.56 (0.19, 1.08) | 0.01 (0.00, 0.02) |
| All Class I TEs | 0.90 (0.00, 2.10) | 0.06 | -0.08 (-0.78, 0.60) | 0.77 (0.77) | 0.48 (0.08, 1.06) | 0.01 (0.00, 0.02) |
| All Class II TEs | 0.68 (-0.22, 1.42) | 0.11 | 0.29 (-0.20, 0.84) | 0.27 (0.60) | 0.29 (0.10, 0.55) | 0.01 (0.00, 0.01) |
| All TEs (Class I + II) | 1.50 (0.73, 2.34) | <0.01 | 0.15 (-0.35, 0.67) | 0.55 (0.63) | 0.28 (0.10, 0.55) | 0.01 (0.00, 0.01) |
| All repeats | 3.18 (2.31, 4.15) | <5e-04 | 0.26 (-0.31, 0.86) | 0.35 (0.60) | 0.38 (0.11, 0.74) | 0.01 (0.00, 0.01) |

The reference levels for fixed factors were as follows: ‘is.desiccating’ = 0.

Results are based on 170 observations of TE length from 34 sample IDs.

pMCMC is defined as twice the posterior probability that the estimate is negative or positive (whichever probability is smallest).

*P*-values are adjusted to account for multiple testing using the Benjamini-Hochberg False Discovery Rate (FDR) method.

**Table 2.** No evidence for an effect of desiccation on TE load across protostomes.

| Test | Fixed effects | | | | Random effects | |
| --- | --- | --- | --- | --- | --- | --- |
|  | Intercept (95% CI) | pMCMC | ‘is.desiccating’ (95% CI) | pMCMC (FDR adjusted) | ID (phylogeny) (95% CI) | Residual (95% CI) |
| DNA transposons | 0.95 (0.17, 1.70) | 0.01 | -0.22 (-0.70, 0.24) | 0.35 (0.49) | 1.27 (0.79, 1.80) | 0.01 (0.00, 0.01) |
| Rolling circles | -2.37 (-3.51, -1.30) | <5e-04 | 0.38 (-0.30, 1.09) | 0.27 (0.47) | 2.67 (1.52, 4.06) | 0.02 (0.00, 0.06) |
| PLEs | -1.97 (-3.47, -0.52) | 0.01 | -0.67 (-1.58, 0.22) | 0.15 (0.36) | 4.72 (2.99, 6.78) | 0.01 (0.00, 0.03) |
| LTRs | 0.52 (-0.32, 1.32) | 0.22 | -0.63 (-1.23, -0.10) | **0.02** (0.28) | 1.53 (0.52, 2.58) | 0.05 (0.00, 0.12) |
| LINEs | 0.69 (-0.16, 1.65) | 0.14 | -0.42 (-0.96, 0.18) | 0.16 (0.36) | 1.85 (1.17, 2.62) | 0.00 (0.00, 0.01) |
| SINEs | -4.75 (-6.82, -3.01) | <5e-04 | 0.03 (-1.36, 1.30) | 0.95 (0.98) | 7.57 (3.64, 12.49) | 0.43 (0.15, 0.70) |
| Satellite | -3.81 (-4.97, -2.60) | <5e-04 | -0.89 (-1.89, -0.01) | **0.05** (0.28) | 3.11 (0.98, 5.74) | 0.49 (0.21, 0.82) |
| Simple | 0.54 (-0.09, 1.22) | 0.10 | -0.23 (-0.66, 0.24) | 0.30 (0.47) | 1.10 (0.66, 1.58) | 0.00 (0.00, 0.01) |
| Low complexity | -1.22 (-1.77, -0.69) | <5e-04 | 0.00 (-0.38, 0.33) | 0.98 (0.98) | 0.66 (0.40, 0.93) | 0.00 (0.00, 0.01) |
| Unclassified | 2.62 (1.90, 3.34) | <5e-04 | 0.32 (-0.13, 0.82) | 0.17 (0.98) | 1.22 (0.76, 1.79) | 0.01 (0.00, 0.02) |
| All Class I TEs | 1.70 (0.94, 2.41) | <5e-04 | -0.46 (-0.92, 0.03) | 0.06 (0.36) | 1.23 (0.77, 1.80) | 0.01 (0.00, 0.02) |
| All Class II TEs | 1.02 (0.33, 1.70) | 0.01 | -0.06 (-0.56, 0.32) | 0.77 (0.28) | 1.05 (0.66, 1.48) | 0.01 (0.02, 0.01) |
| All TEs (Class I + II) | 2.39 (1.77, 3.03) | <5e-04 | -0.27 (-0.64, 0.14) | 0.18 (0.90) | 0.91 (0.55, 1.29) | 0.01 (0.00, 0.01) |
| All repeats | 3.48 (2.94, 4.00) | <5e-04 | 0.07 (-0.25, 0.42) | 0.68 (0.87) | 0.60 (0.34, 0.88) | 0.01 (0.00, 0.02) |

The reference levels for fixed factors were as follows: ‘is.desiccating’ = 0.

Results are based on 295 observations of TE length from 59 sample IDs.

pMCMC is defined as twice the posterior probability that the estimate is negative or positive (whichever probability is smallest).

*P*-values are adjusted to account for multiple testing using the Benjamini-Hochberg False Discovery Rate (FDR) method. Tests with marginal significance for the effect of desiccation prior to FDR adjustment (LTRs and Satellite) are marked in bold.
