# Supplementary material for: Evolutionary dynamics of transposable elements in bdelloid rotifers
Source: eLife. 2021 Feb 5;10:e63194. doi: 10.7554/eLife.63194 (PMC7943196; doi:10.7554/eLife.63194)
Supplement: Figure 4—source data 2. [file elife-63194-fig4-data2.docx]

**Figure 4—figure supplement 3.** Analysis of recombination in LTR-tag presence/absence data.

| Species^1^ | *n* | LTR loci^2^ | ${\bar{\boldsymbol{r}}}_{\boldsymbol{d}}$^3^ | *p* | Consistency index | Frequency of sex^4^ | | |
| --- | --- | --- | --- | --- | --- | --- | --- | --- |
|  |  |  |  |  |  | Median | lower 95% | upper 95% |
| Rg | 5 | 16 | 0.81 | 0.001 | 1.0 | 8.57x10^-06^ | 3.68x10^-07^ | 0.0019169 |
| Rs | 5 | 5 | 0.50 | 0.007 | 1.0 | 1.15 x10^-05^ | 3.86x10^-07^ | 0.01517881 |

^1^ Species codes: **Rg**, *Rotaria magnacalcarata*; **Rs**, *R. socialis*.

^2^ Number of LTR-tag loci that were parsimony informative, i.e. no uniform or single variant individual within the sample.

^3^ $\bar{r}_{d}$ is the modified index of association by Agapow and Burt (2001), *p* is probability of obtaining a value this high in a permutated dataset, i.e. with full out-crossing and recombination, calculated with the ‘ia()’ function in the R package poppr.

^4^ Frequency of sex was calculated by Approximate Bayesian Computation against 50,000 simulated datasets generated by the ‘FacSexCoalescent’ simulator of Hartfield et al. (2018). Simulation parameters: population size 10000, mutation rate 0.001, 900 sites, cross-over rate 2.224694e-06, yielding scaled cross-over rate of 40, frequency of sexual versus asexual reproduction log-uniform prior ranging from 10^-6.5^ to 0.999999. From the simulation output we extracted informative sites (i.e. removing uniform or sites with just a single variant individual) matching the number of informative sites in the observed data (selected to be spread out, i.e. unlinked, along the simulated chromosome). ABC computation used $\bar{r}_{d}$ and Consistency Index as two statistics for inferences, and tolerance 0.05 with the ‘rejection’ method in the ‘abc()’ function in the abc package in R. Posterior distribution is summarized by the median value of the frequency of sex and the upper and lower 95% highest posterior density limits.
