# Supplementary material for: Evolutionary dynamics of transposable elements in bdelloid rotifers
Source: eLife. 2021 Feb 5;10:e63194. doi: 10.7554/eLife.63194 (PMC7943196; doi:10.7554/eLife.63194)
Supplement: Figure 5—source data 2. [file elife-63194-fig5-data2.docx]

**Figure 5—figure supplement 2. Identification of putative telomeric repeats in rotifer genomes.** The telomeric repeat hexamer ‘TGTGGG’ is identified from *A. vaga* but may differ between species. For example, in the bdelloid species *Philodina roseola* it is known to be ‘TGAGGG’, with ‘TGTGGG’ found as a less-frequent variant [7]. In other cases, however, the telomeric repeat may be conserved even across large evolutionary distances (<http://telomerase.asu.edu/sequences_telomere.html>). The telomeric repeat in monogononts is not currently known. Thus, to test if the telomeric repeat sequence has diverged substantially in monogononts, we identified the most frequent G-rich hexamers that did not overlap with any predicted CDS (i.e. discounting any biases in hexamer frequency that may be due to amino acid composition) in the *B. calyciflorus* PSC1 genome assembly using BBTools ‘kmercountexact’ with the parameters ‘k=6 mincount=5’. Visual inspection of the resulting output revealed the hexamers ‘TGTGGG’, ‘TGGTGG’ and ‘TTGGGG’ to be the most frequent and with similar occurrence in the genome (13,499, 13,076 and 16,357 exact matches to non-CDS regions, respectively; see Table below). for comparison, the equivalent counts in the *A. vaga* (Av2013) genome were ‘TGTGGG’ = 58,964, ‘TGGTGG’ = 36,269 and ‘TTGGGG’ = 9720, while in the distantly related bdelloid species *D. carnosus* DCAR706 the counts were ‘TGTGGG’ = 91,200, ‘TGGTGG’ = 123,684 and ‘TTGGGG’ = 74,904. Thus, in lieu of any substantial differences in hexamer frequency within species that might indicate a clear alternative candidate for *B. calyciflorus* (or *D. carnosus*), we decided to include only the frequency of occurrence of the known telomeric repeat ‘TGTGGG’ in all samples.

| Species | Genome | Frequency of repeat | | | Ratio |
| --- | --- | --- | --- | --- | --- |
|  |  | TGTGGG | TGGTGG | TTGGGG |  |
| *A. vaga* | Av2013 | 58,964 | 36,269 | 9720 | 1.0:0.6:0.2 |
| *B. calyciflorus* | PSC1 | 13,499 | 13,076 | 16,357 | 1.0:1.0:1.2 |
| *D. carnosus* | DCAR706 | 91,200 | 123,684 | 74,904 | 1.0:1.4:0.8 |
