# Supplementary material for: Evolutionary dynamics of transposable elements in bdelloid rotifers
Source: eLife. 2021 Feb 5;10:e63194. doi: 10.7554/eLife.63194 (PMC7943196; doi:10.7554/eLife.63194)
Supplement: Figure 5—source data 3. [file elife-63194-fig5-data3.docx]

**Figure 5—source data 3.** Posterior mean and 95% credible intervals for the effects of asexuality (model levels: monogonont = 0, bdelloid = 1; **Table 1**) and desiccation ability (model levels: nondesiccating = 0, desiccating = 1; **Table 2**) on the presence of a number of three genomic features (defined as ‘other genes’; ‘other TEs’; and ‘telomeric repeats’; see main text for details) from a MCMCglmm Gaussian model. The response variable is defined as the (log) number of bases occupied by each feature in a 50 kb window around putative retroelements from the three major subfamilies LINEs, LTRs and PLEs, classified based on their phylogenetic position (see **Figure 5A** and main text).

A model was also run to test for significant differences in the density of the same three genomic features between BUSCO genes (metazoan set, *n* = 978) and TEs (model levels: TE = 0, BUSCO = 1) (**Table 3**).

**Table 1.** No significant effect of asexuality on gene density surrounding genome features.

| Feature | TE type | Fixed effects | | | | Random effects | |
| --- | --- | --- | --- | --- | --- | --- | --- |
|  |  | Intercept (95% CI) | pMCMC | ‘is.bdelloid’ (95% CI) | pMCMC | ID (phylogeny) (95% CI) | Residual (95% CI) |
| Other genes | LTRs | 5.62 (-42.12, 59.41) | 0.85 | -2.11 (-69.03, 67.47) | 0.95 | 645.6 (270.4, 1146) | 11.21 (10.70, 11.78) |
|  | LINEs | 5.92 (-32.33, 46.34) | 0.77 | -1.51 (-57.41, 49.96) | 0.97 | 448 (184.2, 770.6) | 13.94 (13.27, 14.60) |
| Other TEs | LTRs | 6.92 (-31.70, 44.82) | 0.73 | 0.23 (-50.68, 53.09) | 0.97 | 388.7 (205.6, 636.4) | 1.64 (1.55, 1.71) |
|  | LINEs | 7.16 (-21.22, 36.69) | 0.64 | 0.23 (-41.42, 38.75) | 0.97 | 273.8 (139.6, 438.1) | 1.643 (1.56, 1.72) |
| Telomeric repeats | LTRs | 1.26 (-15.02, 16.09) | 0.87 | -0.06 (-19.245, 21.61) | 0.99 | 62.11 (17.29, 118.1) | 2.50 (2.39, 2.63) |
|  | LINEs | 0.97 (-13.95, 15.17) | 0.88 | 0.86 (-18.98, 20.78) | 0.99 | 60.78 (16.57, 113.7) | 2.50 (2.38, 2.62) |

The reference levels for fixed factors were as follows: ‘is.bdelloid’ = 0.

Results are based on 44,069 observations of gene, TE and telomeric density from 31 sample IDs.

pMCMC is defined as twice the posterior probability that the estimate is negative or positive (whichever probability is smallest).

**Table 2.** No significant effect of desiccation on TE density surrounding genome features.

| Feature | TE type | Fixed effects | | | | Random effects | |
| --- | --- | --- | --- | --- | --- | --- | --- |
|  |  | Intercept (95% CI) | pMCMC | ‘is.bdelloid’ (95% CI) | pMCMC | ID (phylogeny) (95% CI) | Residual (95% CI) |
| Other genes | PLEs | 4.68 (-11.01, 20.58) | 0.55 | -1.25 (-11.05, 8.56) | 0.79 | 109.5 (45.33, 193.3) | 11.35 (10.89, 11.85) |
|  | LTRs | 4.11 (-17.62, 27.71) | 0.71 | -0.04 (-15.88, 13.30) | 0.99 | 234.9 (92.45, 407.7) | 11.21 (10.66, 11.74) |
|  | LINEs | 5.49 (-13.45, 25.96) | 0.57 | -1.44 (-13.75, 10.47) | 0.83 | 173.4 (69.4, 311.1) | 13.99 (13.26, 14.67) |
| Other TEs | PLEs | 6.81 (-7.49, 20.54) | 0.35 | 0.21 (-8.75, 9.71) | 0.96 | 97.24 (52.26, 159.7) | 1.29 (1.23, 1.34) |
|  | LTRs | 6.44 (-12.81, 23.30) | 0.47 | -0.01 (-11.61, 10.99) | 0.99 | 146.8 (76.06, 239.6) | 1.64 (1.56, 1.72) |
|  | LINEs | 6.77 (-9.02, 22.01) | 0.41 | -0.20 (-10.15,10.35) | 0.96 | 111.1 (60.11, 180.3) | 1.28 (1.22, 1.35) |
| Telomeric repeats | PLEs | 0.65 (-5.50, 6.19) | 0.81 | 1.22 (-2.39, 5.05) | 0.48 | 14.21 (4.491, 27.12) | 3.47 (3.26, 3.54) |
|  | LTRs | 0.23 (-6.63, 6.61) | 0.96 | 1.33 (-3.36, 5.30) | 0.51 | 20.44 (5.64, 38.24) | 2.51 (2.39, 2.64) |
|  | LINEs | 0.36 (-6.97, 7.16) | 0.91 | 1.69 (-2.73, 6.18) | 0.44 | 21.91 (7.70, 43.00) | 2.65 (2.52, 2.79) |

The reference levels for fixed factors were as follows: ‘is.desiccating’ = 0.

Results are based on 41,708 observations of gene, TE and telomeric density from 29 sample IDs.

pMCMC is defined as twice the posterior probability that the estimate is negative or positive (whichever probability is smallest).

**Table 3.** Significant differences in the genomic context of BUSCO genes versus TEs generally.

| Feature | Fixed effects | | | | Random effects | |
| --- | --- | --- | --- | --- | --- | --- |
|  | Intercept (95% CI) | pMCMC | ‘is.bdelloid’ (95% CI) | pMCMC | ID (phylogeny) (95% CI) | Residual (95% CI) |
| Other genes | 3.51 (-13.80, 21.27) | 0.70 | 5.81 (5.76, 5.85) | **<5e-04** | 184.3 (94.70, 289.1) | 4.51 (4.45, 4.57) |
| Other TEs | 8.19 (-16.30, 33.31) | 0.50 | -3.11 (-3.16, -3.06) | **<5e-04** | 370.8 (190.00, 586.6) | 5.04 (4.98, 5.12) |
| Telomeric repeats | 1.35 (-14.27, 15.70) | 0.86 | 0.45 (0.42, 0.48) | **<5e-04** | 148.7 (83.49, 236.9) | 2.14 (2.12, 2.17) |

The reference levels for fixed factors were as follows: ‘is.bdelloid’ = 0.

Results are based on 132,207 observations gene, TE and telomeric density from 31 sample IDs.

pMCMC is defined as twice the posterior probability that the estimate is negative or positive (whichever probability is smallest).
