# Supplementary material for: Evolutionary dynamics of transposable elements in bdelloid rotifers
Source: eLife. 2021 Feb 5;10:e63194. doi: 10.7554/eLife.63194 (PMC7943196; doi:10.7554/eLife.63194)
Supplement: Figure 5—source data 4. [file elife-63194-fig5-data4.docx]

**Figure 5—source data 4.** Mean and standard deviation (SD) values for the span of features ‘other genes’, ‘other TEs’ and ‘telomeric repeats’ occurring in 50 kb windows around genes of type BUSCO, LINE, LTR or PLE, averaged across monogononts versus bdelloids (**Table 1**) and desiccating versus nondesiccating bdelloids (**Table 2**). Values are in kb.

**Table 1.** Bdelloids vs monogononts.

|  |  | Other genes | Other TEs | Telomeric repeats |
| --- | --- | --- | --- | --- |
| Monogononts | BUSCO | 12.29 ± 8.10 | 1.30 ± 1.41 | 0.01 ± 0.01 |
|  | LINEs | 4.09 ± 5.52 | 2.66 ± 2.16 | 0.01 ± 0.01 |
|  | LTRs | 2.75 ± 3.86 | 2.22 ± 2.03 | 0.003 ± 0.004 |
|  | PLEs | 2.43 ± 3.50 | 2.72 ± 2.36 | 0.01 ± 0.01 |
| Bdelloids | BUSCO | 12.36 ± 8.99 | 0.64 ± 1.52 | 0.02 ± 0.04 |
|  | LINEs | 2.27 ± 4.41 | 3.28 ± 2.92 | 0.03 ± 0.06 |
|  | LTRs | 1.19 ± 2.95 | 3.96 ± 3.77 | 0.02 ± 0.05 |
|  | PLEs | 1.01 ± 2.56 | 4.14 ± 7.03 | 0.03 ± 0.07 |

**Table 2.** Desiccating vs nondesiccating bdelloids.

|  |  | Other genes | Other TEs | Telomeric repeats |
| --- | --- | --- | --- | --- |
| Nondesiccating | BUSCO | 11.87 ± 8.63 | 0.44 ± 1.07 | 0.02 ± 0.03 |
|  | LINEs | 2.25 ± 4.35 | 3.27 ± 2.61 | 0.02 ± 0.04 |
|  | LTRs | 0.97 ± 2.71 | 3.39 ± 3.19 | 0.01 ± 0.03 |
|  | PLEs | 0.85 ± 2.08 | 3.30 ± 6.37 | 0.02 ± 0.04 |
| Desiccating | BUSCO | 12.64 ± 9.18 | 0.74 ± 1.71 | 0.03 ± 0.04 |
|  | LINEs | 2.30 ± 4.50 | 3.30 ± 3.37 | 0.03 ± 0.07 |
|  | LTRs | 1.40 ± 3.15 | 4.53 ± 4.18 | 0.03 ± 0.07 |
|  | PLEs | 1.15 ± 2.92 | 4.92 ± 7.50 | 0.04 ± 0.09 |
