# Supplementary material for: Evolutionary dynamics of transposable elements in bdelloid rotifers
Source: eLife. 2021 Feb 5;10:e63194. doi: 10.7554/eLife.63194 (PMC7943196; doi:10.7554/eLife.63194)
Supplement: Figure 6—source data 2. [file elife-63194-fig6-data2.docx]

**Figure 6—source data 2.** Posterior mean and 95% credible intervals for the effects of asexuality (model levels: monogonont = 0, bdelloid = 1; **Table 1**) and desiccation ability (model levels: nondesiccating = 0, desiccating = 1; **Table 2**) on (log) TE length from a MCMCglmm Gaussian model. Lengths were averaged within TE superfamilies, according to the TE classification system implemented in RepeatMasker.

**Table 1.** No evidence for significant TE length differences between monogononts and bdelloids.

| Test | Fixed effects | | | | Random effects | |
| --- | --- | --- | --- | --- | --- | --- |
|  | Intercept (95% CI) | pMCMC | ‘is.bdelloid’ (95% CI) | pMCMC | ID (phylogeny) (95% CI) | Residual (95% CI) |
| DNA transposons | 4.63 (3.93, 5.35) | <5e-04 | 0.33 (-0.57, 1.31) | 0.45 | 0.13 (0.03, 0.27) | 0.75 (0.71, 0.78) |
| LTRs | 0.75 (0.71, 0.78) | <5e-04 | 0.16 (-0.80, 1.05) | 0.66 | 0.12 (0.00,0.40) | 1.08 (0.93, 1.23) |
| LINEs | 4.85 (4.36, 5.33) | <5e-04 | 0.26 (-0.38, 0.91) | 0.34 | 0.06 (0.00, 0.18) | 1.12 (1.03, 1.22) |

The reference levels for fixed factors were as follows: ‘is.bdelloid’ = 0.

Results are based on 4413 observations of TE length from 42 sample IDs.

pMCMC is defined as twice the posterior probability that the estimate is negative or positive (whichever probability is smallest).

**Table 2.** No evidence for significant TE length differences between desiccating and nondesiccating bdelloids.

| Test | Fixed effects | | | | Random effects | |
| --- | --- | --- | --- | --- | --- | --- |
|  | Intercept (95% CI) | pMCMC | ‘is.desiccating’ (95% CI) | pMCMC | ID (phylogeny) (95% CI) | Residual (95% CI) |
| DNA transposons | 4.92 (4.53, 5.34) | <5e-04 | 0.06 (-0.19, 0.34) | 0.61 | 0.07 (0.01, 0.15) | 0.79 (0.75, 0.84) |
| PLEs | 5.80 (5.33, 6.21) | <5e-04 | 0.06 (-0.29, 0.34) | 0.66 | 0.08 (0.01, 0.21) | 0.07 (0.05, 0.10) |
| LTRs | 5.21 (4.77, 5.66) | <5e-04 | 0.21 (-0.14, 0.54) | 0.20 | 0.06 (0.00, 0.19) | 1.17 (0.99, 1.34) |
| LINEs | 5.14 (4.78, 5.49) | <5e-04 | -0.03 (-0.29, 0.24) | 0.84 | 0.04 (0.00, 0.12) | 1.23 (1.11, 1.35) |

The reference levels for fixed factors were as follows: ‘is.desiccating’ = 0.

Results are based on 3646 observations of TE length from 34 sample IDs.

pMCMC is defined as twice the posterior probability that the estimate is negative or positive (whichever probability is smallest).
