# Supplementary material for: Evolutionary dynamics of transposable elements in bdelloid rotifers
Source: eLife. 2021 Feb 5;10:e63194. doi: 10.7554/eLife.63194 (PMC7943196; doi:10.7554/eLife.63194)
Supplement: Figure 8—source data 1. [file elife-63194-fig8-data1.docx]

**Figure 8—source data 1.** CAFE model fitting birth rate model to gene family evolution of RNAi pathways.

|  |  | Log likelihood | *P* shift in birth rate | Average birth rate |
| --- | --- | --- | --- | --- |
| Combined model | Argo | -240.21 | <0.0001 | 0.0018 |
|  | Dicer | -57.06 | 0.999 | 0.0018 |
|  | RdRP | -390.25 | <0.0001 | 0.0018 |
|  |  |  |  |  |
| Separate models | Argo | -239.8 | <0.0001 | 0.0018 |
|  | Dicer | -52.66 | 0.999 | 0.00078 |
|  | RdRP | -390.18 | <0.0001 | 0.0019 |
